# Empathy: A clue for prosocialty and driver of indirect reciprocity

**DOI:** 10.1371/journal.pone.0255071

**Published:** 2021-08-12

**Authors:** Frauke von Bieberstein, Andrea Essl, Kathrin Friedrich

**Affiliations:** Institute of Organization and Human Resource Management, University of Bern, Bern, Switzerland; Teesside University, UNITED KINGDOM

## Abstract

Indirect reciprocity has been proposed to explain prosocial behavior among strangers, whereby the prosocial act is returned by a third party. However, what happens if the prosocial act cannot be observed by the third party? Here, we examine whether empathy serves as a clue for prosociality and whether people are more generous toward more empathetic people. In a laboratory study, we measured prosocial behavior as the amount sent in the dictator game and empathy based on the Interpersonal Reactivity Index (IRI). By using an incentivized task, we find that people believe that more empathetic participants send more money in the dictator game. Thus, people see empathy as a clue for prosocial behavior. Furthermore, in a second dictator game, participants indirectly reciprocate by sending more money to more empathetic recipients. Therefore, we suggest that empathy can replace a reputation derived from observable prosocial behavior in triggering indirect reciprocity.

## Introduction

Prosocial behavior—taking a costly action that benefits others—is an important building block of society [1]. Many people act prosocially, not only toward kin, but also toward strangers [2]. For instance, people donate money [3], share food [4], help a stranger in need [5], and contribute to common-pool resources [6]. Prosocial behavior can be stable due to reciprocity, such that an action is returned either directly by the recipient or indirectly by a third party [7, 8]. Given that many interactions in modern society are one-time encounters between strangers, indirect reciprocity has received substantial interest in the last decade. There is ample evidence of indirect reciprocity in the lab and in the field, for instance, in trust games [9], dictator games [8], and in a more applied context, on an online platform for free services [10].

Psychologists consider empathy, which is an emotional response to experiences of another being, to be among the most important motives for prosocial behavior. Several studies support the close relation of empathy and prosociality. They postulate that empathy elicits prosocial (altruistic) behavior [11–14], a suggestion known as the empathy-altruism hypothesis [15–17].

To sum up, people are directly and indirectly reciprocal to others who have acted prosocially, and researchers have shown that prosocial behavior and empathy are positively related. In this article, we combine these two strands of literature to examine whether empathy triggers a similar reciprocal response as prosocial behavior. This could be the case if empathy serves as a clue for prosociality. Thus, we tackle the following research questions: 1) Does empathy serve as a clue for prosocial behavior? 2) Is empathy rewarded even in the absence of information about previous actions? We address these questions using an incentivized laboratory study.

The laboratory study consisted of four stages: First, we evaluated participants’ prosocial behavior in a standard dictator game. Second, we measured empathy based on the German version of IRI [18, 19]. Third, we assessed whether people expect that more empathetic individuals behave more prosocially. Participants were paid for correctly guessing how much money another participant passed in the standard dictator game of stage 1, given his or her particular level of empathy as measured in stage 2. Fourth, participants played a conditional dictator game. In contrast to the standard dictator game, they had to indicate how much money they sent for every possible empathy level of the recipient. The design allowed us to examine whether empathy is perceived as a clue for prosocial behavior (stage 3) and whether people indirectly reciprocate higher levels of empathy (stage 4) in an incentivized environment. We find that empathy serves as a clue for prosocial behavior because people believe that more empathetic individuals send more money in the dictator game. Furthermore, dictators act more prosocially toward people who are more empathetic (stage 4).

Our study makes several important contributions to the literature. First, we provide both of the above findings. Second, considering the relation between these findings is of high interest, as it allows us to uncover a potential mechanism: Empathetic people are perceived to be more prosocial, which might trigger indirect reciprocal behavior toward them. Finally, the results contribute to the research on cooperation and indirect reciprocity [2, 7]. In previous studies, indirect reciprocity was based on reputation and thus, on observable prosocial behavior [1, 8, 20]. However, given that many interactions in modern society are encounters between strangers, there is often no information about previous behavior, and in turn, no reputation to build on. Instead of gathering information about a person’s previous prosocial acts, it might be easier to assess his or her level of empathy. In fact, research has shown that people do remarkably well in identifying individuals characterized by high and low empathy from a brief sample of behavior based on scant or fleeting information [21]. Our results suggest that empathy can substitute for reputation derived from past prosocial behavior. Consequently, indirect reciprocity can occur even without knowing someone’s reputation for prosocial behavior.

### Related literature and hypotheses

Prosocial behavior is defined as “voluntary, intentional behavior that results in benefits for another person” [15, p. 92]. Prosocial behavior is affected by dispositional factors (i.e., stable personal characteristics that predispose an individual to behave prosocially) as well as by situational factors (i.e., stimulis in an individual’s environment that promote prosocial behavior) [22]. Similarly, researchers distinguish between measures of dispositional empathy, in which empathy is defined as a stable character trait of a person, and situational empathy, which is understood as an individual’s empathetic response in a specific situation.

Various laboratory experiments have revealed a positive relationship between empathy as a situational factor and prosocial behavior [11, 12, 15, 16, 23, 24]. For example, Stocks, Lishner, and Decker [23] induced empathy and found that empathically aroused participants intend to spend more time to help a person in need. Similarly, Barraza and Zak [11] and Klimecki, Mayer, Jusyte, Scheff, and Schönenberg [12] stimulated empathy showing that higher levels of empathy are associated with higher monetary offers in the ultimatum game and the dictator game, respectively. The relationship between empathy as a dispositional factor and prosocial behavior is less clear. Some studies detected statistically significant but weak correlations [25–27], and others found none [28, 29]. Closely related to our research, Edele, Dziobek, and Keller [27] and Franzen, Mader, and Winter [13] examined the relationship between the stable characteristic empathy based on the IRI [18] and giving in the dictator game. The IRI measures four dimensions of dispositional empathy: *empathic concern*, *personal distress*, *fantasy*, and *perspective taking* [18]. Edele, Dziobek, and Keller [27] elicited only two of the dimensions and found that the correlation between empathy and altruism is statistically significant only for *empathic concern*, whereas it is not for *perspective taking*. In contrast, Franzen, Mader, and Winter [13] identified a positive and statistically significant correlation for all dimensions except *fantasy*.

Based on the above findings, we hypothesize:

**Hypothesis 1**: *People believe that more empathetic people send more money in the dictator game*.

Moreover, there is experimental evidence that people alter their behavior in response to empathy. Brooks, Dai, and Schweitzer [30] showed that superfluous apologies signal empathy and in turn, increase trust in the apologizer. Von Bieberstein, Goette, and Guentner [31] conducted a field experiment with a roadside assistance provider and concluded that increasing the level of empathy expressed during a service encounter increases customers’ loyalty to the decision to have their cars repaired at the recommended mechanic.

The theory of indirect reciprocation formalizes the pervasive phenomenon that prosocial acts are returned, not by the recipient, but by a third party [2, 7, 32]. Such behavior is ubiquitous in economic life, although it is neither rational nor stable as it increases others’ payoffs at one’s own expense. Two indirect reciprocity mechanisms can be differentiated: generalized indirect reciprocity, which is based on recent positive experience, and social indirect reciprocity, which is based on reputation [1]. Generalized indirect reciprocity implies that kind (or unkind) acts are reciprocated toward a third party (if A helps B, then B helps C). Social indirect reciprocity means that a kind (or unkind) act is reciprocated by a third party (if A helps B, then C helps A) [20].

There is broad experimental evidence supporting the existence of generalized indirect reciprocity (e.g., [8, 9, 20, 33–35]) and social indirect reciprocity (e.g., [8, 20, 32, 36, 37]). Most closely related to the present study are the experiments in [20] and [8] because they also examine social indirect reciprocity in settings where reciprocal behavior cannot be explained by strategic motives. Indirect reciprocation in the absence of any strategic motives is defined as strong or pure indirect reciprocity [38, 39]. Stanca [20] built on a gift exchange game played in two stages to investigate generalized indirect reciprocity and social indirect reciprocity. The results suggested that generalized indirect reciprocity is statistically significantly stronger than social indirect reciprocity and direct reciprocity. Herne, Lappalainen, and Kestilä-Kekkonen [8] tested indirect social reciprocity in a dictator game where the second-round dictators reacted to the first-round dictator’s behavior when he or she had not taken part in the first round him- or herself but was informed about the dictator’s behavior. Again, dictators acted more prosocially in stage 2 the more prosocially the beneficiary had acted in stage 1.

In contrast to our study, participants in previous experiments on social indirect reciprocity were informed about the prosocial behavior of the other person before deciding about their own prosocial behavior. This is in line with the reasoning that reputation must be observable, for example, in terms of traceable behavior, for social indirect reciprocity to be effective [1, 40]. Only if people’s kind actions are noticed can reputation foster prosocial behavior [40]. In our setting, behavior in the dictator game is not observable. Instead, participants receive information about the beneficiary’s empathy level, which we expect them to perceive as a clue for previous prosocial behavior (see Hypothesis 1). Based on social indirect reciprocity, we hypothesize:

**Hypothesis 2**: *People send more money in the dictator game the more empathetic the recipient is*.

## Materials and methods

**Fig 1:**
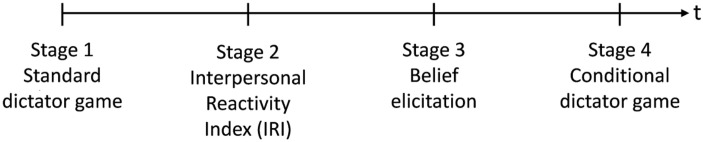
Stages of the study.

We conducted a four-stage laboratory study to analyze whether empathy is perceived as a clue for prosociality and how people adapt their behavior in response. We pre-registered the study with the platform AsPredicted.org with the unique identifying number 2370 and obtained ethical approval from the Faculty of Business Administration, Economics and Social Sciences of the University of Bern. Fig 1 gives an overview of the four stages of the study. An English translation of the original German instructions is available in the S1 Appendix.

In stage 1, we used a one-shot dictator game (DG) to measure prosocial behavior. The DG is the standard decision task to study altruism in behavioral economics [41, 42]. We implemented the dictator game to rule out motives other than the intention to benefit others (e.g., strategic concerns which are often driven by self-interested considerations). In this game, the dictator was endowed with 10 CHF (about 10 USD) and decided how much, if any, of the endowment he or she transferred to an anonymous recipient.

In stage 2 of the study, we measured empathy based on the German version of the Interpersonal Reactivity Index (Saarbrücker Personality Questionnaire) [19]. This validated, self-report psychometric test is commonly used to assess empathy [43–45]. It consists of 16 items that describe social interactions in four dimensions: *perspective taking*, *fantasy*, *empathic concern*, and *distress* (see Table 1). On a 5-point Likert scale (“Never” to “Always”), participants indicated how much each of the 16 statements applied to them. Based on the average score across all items, participants were assigned one out of five possible levels of empathy (very low, low, medium, high, or very high). We used standard mathematical rounding to calculate the average empathy scores. An overview of the empathy levels and the associated average scores is available in the S1 Appendix. Importantly, half of the subjects received the original scale from 1 (“Never”) to 5 (“Always”), while the other half received a reversed scale from 5 (“Never”) to 1 (“Always”). By reversing the number labels, we excluded that the numeric averages instead of the assigned empathy levels drove the behavior in stages 3 and 4. For example, it could be that higher numerical averages, irrespective of their meaning, elicit higher expectations in the belief elicitation stage and/or higher transfers in the conditional dictator game. We find that reversing the number labels of the IRI scale did not affect any of our results.

**Table 1:** The four dimensions of the Interpersonal Reactivity Index (IRI).

Dimension and items		Factor loadings
Perspective taking		
1.	I try to look at everybody’s side of a disagreement before I make a decision.	0.77
2.	I believe that there are two sides to every question and try to look at them both.	0.64
3.	When I am upset with someone, I usually try to put myself in his shoes for a while.	0.48
4.	Before criticizing somebody, I try to imagine how I would feel if I were in their place.	0.68
Fantasy		
5.	I really get involved with the feelings of the characters in a novel.	0.59
6.	After seeing a play or movie, I have felt as though I were one of the characters.	0.58
7.	When I watch a good movie, I can very easily put myself in the place of a leading character.	0.64
8.	When I am reading an interesting story or novel, I imagine how I would feel if the events in the story were happening to me.	0.63
Empathic concern		
9.	I often have tender, concerned feelings for people less fortunate than me.	0.69
10.	When I see someone being taken advantage of, I feel kind of protective toward them.	0.43
11.	I am often quite touched by things that I see happen.	0.51
12.	I would describe myself as a rather soft-hearted person.	0.67
Personal distress		
13.	In emergency situations, I feel apprehensive and ill-at-ease.	0.63
14.	I sometimes feel helpless when I am in the middle of a very emotional situation.	0.62
15.	Being in a tense emotional situation scares me.	0.73
16.	I tend to lose control during emergencies.	0.45
*N*		109
Cronbach’s *α*		0.75

*Notes*: Values indicate factor loadings after the varimax-rotated principal factor analysis

Stage 3 revealed whether empathy is perceived as an indicator of prosocial behavior, i.e., whether people expect that more empathetic people behave more prosocially. Participants were incentivized to truthfully report their beliefs about another randomly chosen participant’s money transfer in the standard dictator game. They conditioned their beliefs on the level of empathy measured in stage 2. More precisely, we used the strategy method [46] such that all participants indicated the amount of money they believed another participant had sent in the standard dictator game in stage 1 for each of the five different levels of empathy as measured by the average of the answers in stage 2. At the end of the experiment, each person was randomly assigend to another participant. This participant’s empathy score and prosocial behavior determined the counterpart’s payment: Guessing correctly paid 10 CHF and deviating by 1 CHF from the actual transfer 5 CHF. For any other deviation, no additional money was paid.

In stage 4, we examined whether people are more generous toward more empathetic people using a conditional dictator game. We adopted the standard dictator game using the strategy method [46] to elicit prosocial behavior conditional on the empathy level of the recipient. Participants decided how much, if any, of their endowment of 10 CHF they sent for each possible empathy level of the recipient. Thus, each subject indicated the amount of money he or she transferred to a recipient characterized by very low, low, medium, high, and very high empathy. Finally, participants provided demographic information on a short questionnaire before being paid.

In stages 1 and 4, we used role uncertainty to collect decisions in the role of the dictator from all subjects. At the end of the study, a random mechanism determined which of the two stages was relevant for payment, in addition to the payment from stage 3. We implemented a perfect-stranger-matching protocol over all stages to avoid reputation concerns. Participants knew that the study consisted of four stages and that they would receive the instructions for each stage only after having completed the previous stage. To avoid strategic concerns, we did not mention in the instructions of stage 2 that payoffs in the second dictator game might depend on participants’ IRI scores. More specifically, knowing that decisions in the second dictator game can be conditioned on participants’ IRI scores, might lead individuals to alter their responses in the questionnaire for strategic reasons rather than revealing their true empathy level. However, this comes with two concerns. First, it could be that participants in later sessions learn from previous participants that higher IRI scores could be preferable. Yet, in our experiment, the IRI scores did not increase with the number of sessions. The Pearson correlation coefficient of *ρ* = 0.014 between the IRI score and the sessions is not statistically significant. Second, it could be that participants thought after the experiment that they would have liked to answer the IRI scale differently if they had known about the second dictator game. Although none of the participants approached us on this, we cannot rule out these thoughts. In this respect, the experiment is in a similar spirit as experiments with surprise restart effects (e.g., [47]) or matching on conditional behavior (e.g., [48]). For a critical discussion on this see [49]. It should be noted that neither the participants nor the experimenters themselves knew at the time of the experiment whether a higher IRI score would really pay off. As we argue in the Discussion section, it could well be that participants are generous towards participants with a similar (and not necessarily a higher) IRI score than oneself. However, we did not find evidence for this type of behavior in the data.

We ran our power analysis based on the results of a pilot study. The pilot study was conducted during class with 49 students of the University of Bern. In this study, we found effect sizes of 0.29 or larger with respect to Hypothesis 1. Based on a two-sided Wilcoxon signed-rank test with a minimum detectable effect size of 0.29, an error probability *α* of 0.05, and a power of 0.80, we required 100 observations.

We conducted the main study with 109 students from various disciplines in the Aare-Lab of the University of Bern between June and September 2019. Subjects were recruited via Sona-Systems, and the study was computerized using z-Tree [50]. Participants were, on average, 24.17 years old (S.D. = 6.05, range = 19–67), and 41% were female. To ensure that all participants understood the dictator game, the belief elicitation stage, and associated payoffs, they could start with the games only after correctly answering control questions. The sessions lasted about 45 minutes, and earnings averaged 17.39 CHF (S.D. = 4.55 CHF, range = 8.00–29.00 CHF), including a show-up fee of 4 CHF.

## Results

**Fig 2:**
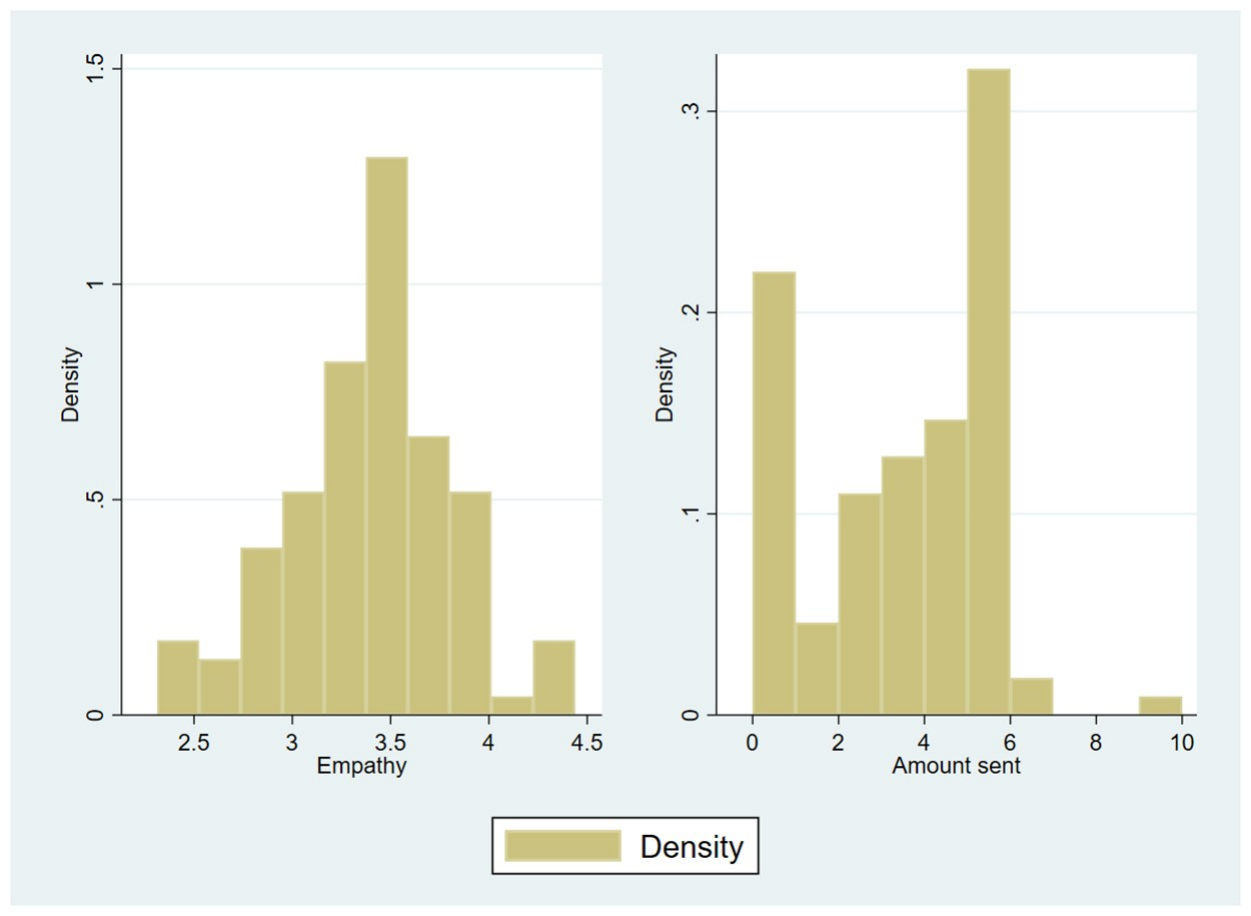
Distribution of empathy and prosocial behavior. Distribution of empathy (left panel) and prosocial behavior measured by the amount sent in the first stage dictator game (right panel).

To assess empathy, we used the 16 items of the IRI shown in Table 1 and performed a principal factor analysis with maxvari rotation. In line with previous research, four subdimensions emerged from our analysis, which are commonly referred to as *perspective taking*, *fantasy*, *empathic concern*, and *personal distress* (see Table 1 for factor loadings). The IRI produced an overall Cronbach’s *α* of 0.75. The average IRI score is 3.38 (S.D. = 0.42, range = 2.31–4.44), and its distribution is shown in the left panel in Fig 2.

In stage 1, participants played the standard dictator game with an endowment of 10 CHF. The amount of money sent serves as a measure of prosocial behavior. On average, subjects transferred 3.05 CHF (i.e., 31% of their endowment) to an anonymous recipient. 22% of the participants acted completely selfishly, and 32% transferred half of the endowment. In the right panel of Fig 2, the distribution of prosocial behavior is summarized. In the sample, we identified a positive relationship between individual empathy and own prosocial behavior. However, this correlation is not statistically significant or only significant at a 10% level if the *personal distress* subdimension of the IRI scale is excluded (see S1 Table). The *personal distress* subdimension is sometimes excluded from analysis because it might measure self-management rather than empathy [13, 19]. To provide reasonable statistical power to detect significant correlation between the level of empathy and the amount sent in the dictator game we would need a higher sample size. The meta-analysis of traits of individual differences and behaviors in experimental games [14] reveals a correlation between empathy and distribution in dictator games of about 0.12. To detect this correlation with a power of at least 0.8 at the 0.05 significant level a sample size of 542 would be required. However, our sample size was determined based on our hypotheses (see Hypothesis 1 and 2) and not on the correlation between an individual’s level of empathy and the amount this individual sent (see section on materials and methods for the detailed power analysis).

**Fig 3:**
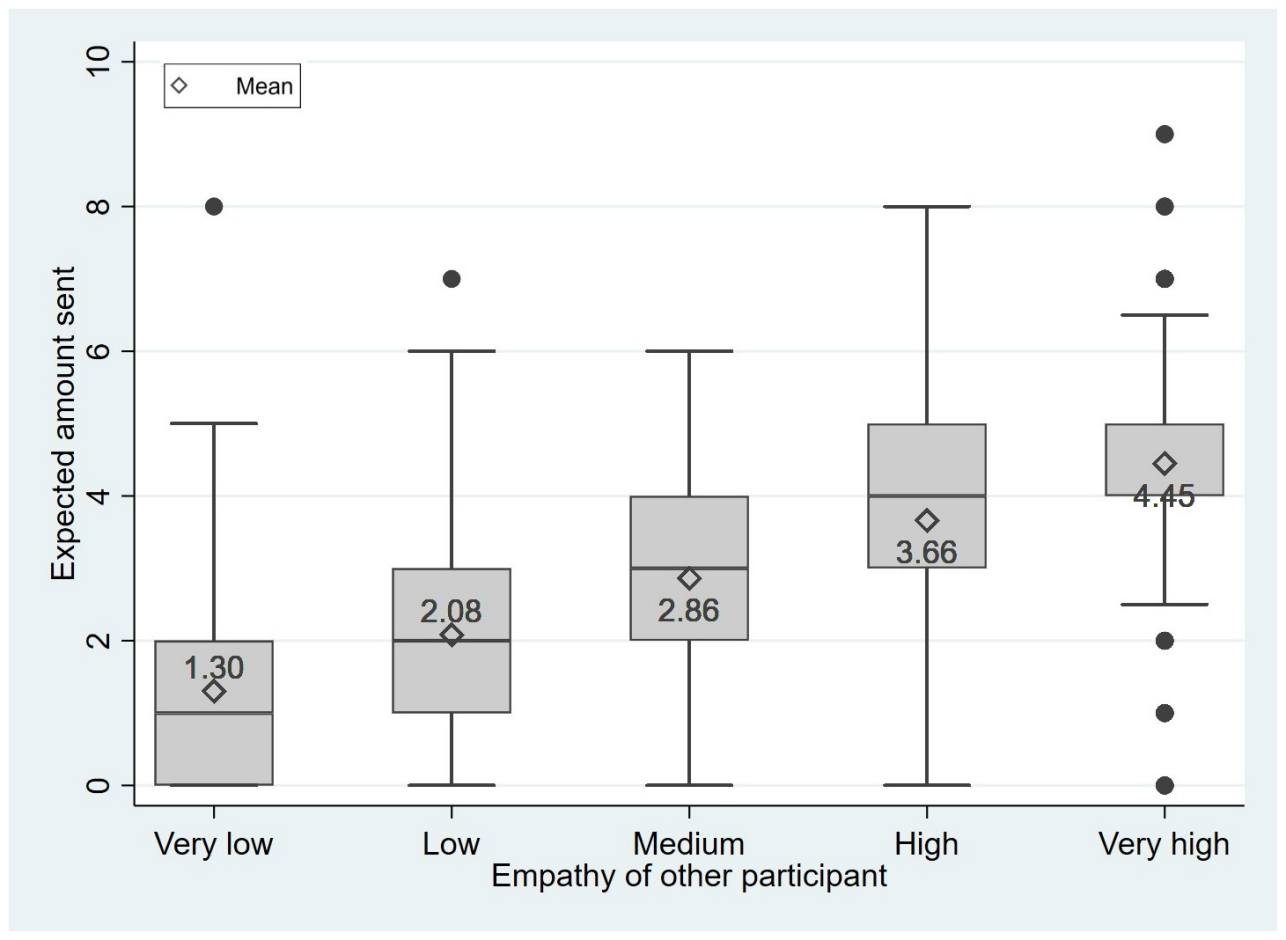
Boxplot of expected prosocial behavior conditional on the empathy of the other participant.

Turning to the first hypothesis, we test whether participants perceive empathy as an indicator of prosocial behavior based on the belief elicitation in stage 3. Fig 3 depicts the expectations that participants formed about the prosociality of others based on the assigned empathy level of that other person. Fig 3 reveals that the participants’ expectations about the amounts sent in the dictator game increased in the empathy level of the other participant. If a dictator had a very low level of empathy, participants expected an average transfer of 1.30 CHF (S.D. = 1.39) of him or her. For a low level of empathy, expectations were 2.08 CHF (S.D. = 1.51), for medium empathy 2.86 CHF (S.D. = 1.55), for high empathy 3.66 CHF (S.D. = 1.72), and for very high empathy 4.45 CHF (S.D. = 1.93). Pairwise comparison revealed that these differences are statistically significant (*p*<0.001 for all pairwise comparisons, Wilcoxon signed-rank tests).

Table 2 shows the results of the corresponding pooled ordinary least squares (OLS) regressions explaining participants’ expectations for others’ amounts sent conditional on empathy. The estimates in model 1 indicate that the higher the empathy level, the more prosocially the others believed the participant to have behaved. Participants expected that sending in the standard dictator game increased by 0.79 CHF for each step of one level on the 5-point empathy scale (i.e., from very low to low, from low to medium, etc.). This means that a person’s empathy is highly predictive of expected prosocial behavior and serves as a clue. Model 2 of Table 2 displays that the own amount sent is also highly predictive of the beliefs formed about others’ prosociality. However, the effect size is smaller than that of the other’s empathy level, which is robust in terms of significance and size. Controlling for gender and age (model 3) does not alter these findings. Thus, we conclude:

**Table 2:** Effect of another participant’s empathy on expected prosocial behavior—Pooled OLS regression.

	Expected amount sent (1)	Expected amount sent (2)	Expected amount sent (3)
Empathy of other participant	0.787***	0.787***	0.787***
	(0.048)	(0.048)	(0.048)
Own amount sent in stage 1		0.444***	0.441***
		(0.068)	(0.068)
Female			-0.208
			(0.213)
Age			0.004
			(0.011)
Constant	0.510***	-0.841***	-0.833***
	(0.165)	(0.197)	(0.310)
Observations	545	545	545
*R* ^2^	0.320	0.539	0.542

*Notes*: The table presents the results of a pooled OLS regression with robust standard errors clustered on the individual level in parentheses. The dependent variable is the expected amount sent measured as beliefs (stage 3). Empathy of other participant indicates every possible level of empathy the other participant can have. Own amount sent in stage 1 is the amount sent of the participant in the standard dictator game (stage 1). Female indicates whether the participant is female (= 1) or not (= 0). Age gives the age of the participant.

*, **, and *** document significance at the 5%, 1%, and 0.1% levels, respectively.

**Result 1**: *People believe that more empathetic people behave more prosocially*.

**Fig 4:**
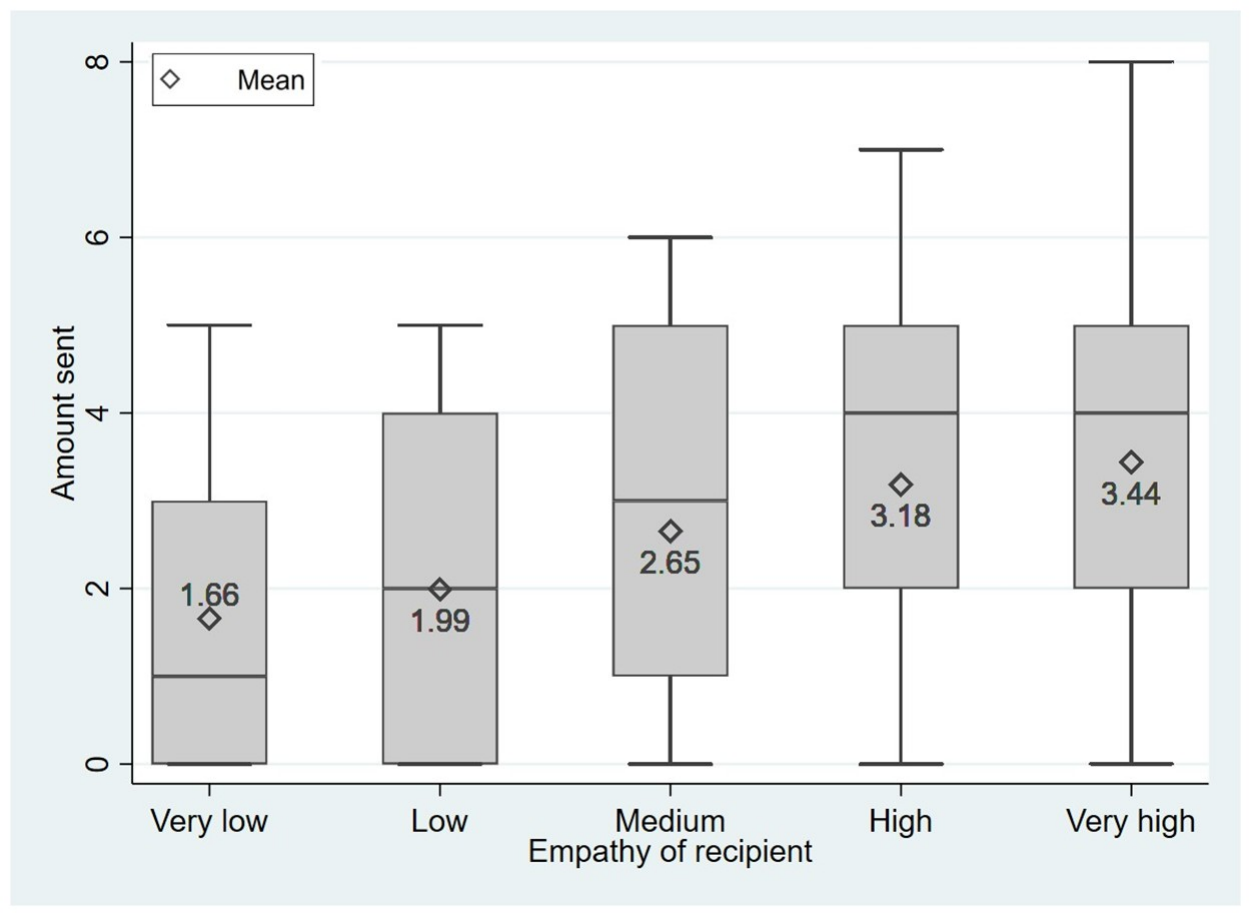
Boxplot of prosocial behavior conditional on the empathy of the recipient.

Next, based on the conditional dictator game results in stage 4, we examine whether people indirectly reciprocate higher empathy with more prosociality. Fig 4 shows a positive relationship between the empathy level of the recipient and the amount sent in the conditional dictator game. Starting from a mean sending of 1.66 CHF (S.D. = 1.89) for very low empathy of the recipient, the mean sending increased to 1.99 CHF (S.D. = 1.82), 2.65 CHF (S.D. = 1.93), 3.18 CHF (S.D. = 1.99), and 3.44 CHF (S.D. = 2.18) for low, medium, high, and very high empathy of the recipient. The differences between all means are statistically significant (*p*<0.001 for all pairwise comparisons, Wilcoxon signed-rank tests).

The results of the pooled OLS regression displayed in Table 3 with amounts sent in the conditional dictator game as the dependent variable confirm the descriptive statistics. Empathy of the recipient affects amounts sent statistically significantly. On average, raising the empathy level of the recipient by one increased the transfer amount by 0.48 CHF in model 1. Furthermore, according to model 2, the own amount sent in the standard dictator game in stage 1 is again highly predictive. That is, participants who sent 1 CHF more in stage 1, transferred, on average, almost 0.69 CHF more in the conditional dictator game. Thus, we support the conclusion by [51] pointing out that altruists indirectly reciprocate stronger than egoists. The effect of the recipients’ empathy is robust in terms of significance and size. This is also the case when we additionally control for gender and age (model 3). Therefore, we find:

**Table 3:** Effect of the recipient’s empathy on prosocial behavior—Pooled OLS regression.

	amount sent (1)	amount sent (2)	amount sent (3)
Empathy of recipient	0.475***	0.475***	0.475***
	(0.045)	(0.045)	(0.046)
Own unconditional amount sent		0.687***	0.678***
		(0.093)	(0.092)
Female			-0.191
			(0.222)
Age			0.028***
			(0.008)
Constant	1.160***	-0.933***	-1.512***
	(0.200)	(0.250)	(0.285)
Observations	545	545	545
*R* ^2^	0.105	0.581	0.591

*Notes*: The table presents the results of a pooled OLS regression with robust standard errors clustered on the individual level in parentheses. The dependent variable is the amount sent in the conditional dictator game (stage 4). Empathy of recipient indicates every possible level of empathy the recipient can have. Own amount sent in stage 1 is the amount sent of the participant in the standard dictator game (stage 1). Female indicates whether the participant is female (= 1) or not (= 0). Age gives the age of the participant.

*, **, and *** document significance at the 5%, 1%, and 0.1% levels, respectively.

**Result 2**: *People behave more prosocially toward more empathetic people*.

## Discussion

In this study, we revisited the relationship between empathy and prosocial behavior. We focused on expectations derived from empathy, as well as on the adaption of one’s own prosocial behavior in response to the empathy level of others. The results reveal that people form beliefs about others’ prosocial behavior based on empathy. Specifically, empathetic people are expected to act more prosocially, i.e., to send more money in the dictator game. Thus, we suggest that revealed empathy is perceived as a clue for prosocial behavior. At the same time, we show that the higher the level of empathy of the recipient, the more generous the dictators. This result supports the social indirect reciprocity perspective [1]. Taken together, the present findings imply that people indirectly reciprocate empathy most likely because they associate higher levels of empathy with a reputation for being prosocial.

We suggest that social indirect reciprocity explains the higher amounts sent in the dictator game in response to a higher level of empathy. This is backed by further analysis, which allows us to exclude social distance and inequality aversion as two alternative explanations. Reducing social distance between dictators and recipients has been shown to raise amounts sent in the dictator game [52–55]. The social distance explanation thus proposes that dictators send on average more money in stage 4 than in stage 1 of our experiment, as learning the self-assessed empathy level of the counterpart lowers social distance. Yet the present data reveal that dictators sent, on average, statistically significantly less when information about the beneficiary’s empathy level was given in stage 4 (Mean = 2.59, S.D. = 1.81) compared to the standard dictator game in stage 1 (Mean = 3.05, S.D. = 2.09) (p<0.001, Wilcoxon signed-rank test). Further, if social distance had been crucial in this study, the matching of empathy levels between the dictator and the recipient would have potentially affected prosocial behavior in the conditional dictator game. However, we did not find that expectations or amounts sent increased significantly if two highly empathetic participants met, and they did not change for two individuals with low empathy, or mixed pairs (see S2 and S3 Tables). In addition, we rule out that inequality aversion [56, 57] drives the positive correlation between the recipients’ level of empathy and the dictator’s prosociality. Inequality aversion would imply higher (lower) amounts sent in the conditional dictator game, if the dictator gives a low (high) amount in stage 1 but expects a highly (hardly) empathetic recipient to have sent a high (low) amount in stage 1. However, we did not find such effects. The results shown in Table 3 suggest that the empathy level of the recipient positively affects the amount sent even though we control for the own amount sent in the standard dictator game. Additionally, we can show that the (absolute) difference between the own amount sent in the standard dictator game and the expected sending of the counterpart does not have a statistically significant effect on prosocial behavior in the conditional dictator game (see S4 Table).

These results provide important behavioral insights. By demonstrating that people derive expectations about an individual’s prosocial behavior from his or her empathy, we identify an additional clue for prosociality beyond gender [58, 59]), and social closeness [60]. Furthermore, the results add to a more comprehensive picture on cooperation initiated by social indirect reciprocity [2, 7]. Previous studies assume that reputation, which results from observable prosocial behavior, is the driver of social indirect reciprocity [1, 40]. We complement this literature by providing compelling evidence that empathy as an observable characteristic can replace reputation for prosociality in eliciting indirect reciprocity. This is particularly relevant for one-time encounters between strangers, as it might be easier to assess a person’s level of empathy than to gather information about his or her prosocial acts.

Some limitations inherent to our study raise open questions and provide avenues for future research. Receiving information about empathy based on the IRI test score might have appeared artificial. In addition, experimenter demand effects might have occurred if participants had expected that reciprocation of empathy was the desired behavior. To minimize such possible experimenter demand effects, we never mention “empathy” in the instructions, but used “average answer on the survey in part 2.” Although, to address these issues, future work could conduct a similar study in a more applied context. A promising first step might be to test the robustness of the pinpointed relationships between empathy and (expected) prosocial behavior based on experiencing empathy instead of mere information (e.g., as in [30, 31]). Similarly, examining whether and how beliefs about prosociality update over the course of interactions, and whether the positive effect of empathy on prosocial behavior persists over time, broadens the applicability to real-world relations as it would no longer be restricted to encounters between strangers. Furthermore, as we focus on social indirect reciprocity, it is open to future research to explore generalized indirect reciprocity and direct reciprocity combined with information about the empathy level of a beneficiary. Moreover, as there is empirical evidence that women portray themselves as more empathetic [61–63] and also in this study, women were statistically significantly more empathetic (*Mean* = 3.56, S.D. = 0.32) than men (*Mean* = 3.26, S.D. = 0.43) based on the IRI score (*p*<0.001, Mann-Whitney rank-sum test). Thus, exploiting the interplay of empathy and gender as a perceived indicator of prosocial behavior might be a fruitful approach to gain a more sophisticated understanding of the foundations of expectations about prosociality. In addition, another relevant direction for future research could be to investigate the role of aversion to disappointing the expectations of empathetic people. Ederer and Schremitzer [64], for example, have shown that a promisor’s aversion to disappointing a promisee’s expectation induces more generosity. Given that people form different expectations about prosocial behavior based on empathy, they might also believe that more empathetic people expect higher returns. Therefore, eliciting dictators’ beliefs about the beneficiary’s expectations would shed more light on the potential reasons for increasing prosociality toward more empathetic people. Finally, if empathy is used as a clue to identify prosocial behaviors and if such a clue is rewarded financially, it could be that selfish individuals try to mimic empathetic clues without the corresponding prosocial behavior. Thus, it would be interesting to explore under which conditions empathy could be an honest signal or not.

## Supporting information

S1 Table Effect of own empathy on prosocial behavior—OLS regression. (PDF)

S2 Table Effect of another participant’s empathy on expected prosocial behavior, type matching—Pooled OLS regression. (PDF)

S3 Table Effect of the recipient’s empathy on prosocial behavior, type matching—Pooled OLS regression. (PDF)

S4 Table Effect of differences between the own amount sent and the expected amount sent of the counterpart on prosocial behavior. (PDF)

S1 Appendix Instructions. (PDF)
