# Supplementary material for: Empathy: A clue for prosocialty and driver of indirect reciprocity
Source: PLoS One. 2021 Aug 12;16(8):e0255071. doi: 10.1371/journal.pone.0255071 (PMC8360368; doi:10.1371/journal.pone.0255071)
Supplement: S1 Table — (PDF) [file pone.0255071.s001.pdf]

S1 Table. Effect of own empathy on prosocial behavior - OLS regression.

	Amount sent (1)	Amount sent (2)	Amount sent (3)	Amount sent (4)
Own empathy	0.585	0.633		
	(0.500)	(0.576)		
Own empathy, restricted index			0.738	0.781
			(0.412)	(0.462)
Female		-0.272		-0.301
		(0.474)		(0.458)
Age		0.021		0.018
		(0.023)		(0.022)
Constant	1.068	0.515	0.367	-0.093
	(1.706)	(1.723)	(1.518)	(1.540)
Observations	109	109	109	109
R^2	0.014	0.022	0.029	0.038

Notes: The table presents the results of an OLS regression with robust standard errors in parentheses. The dependent variable is the amount sent in the standard dictator game (stage 1). Own empathy is the IRI score of the participant (stage 2). Own empathy with restricted index is the IRI score of the participant without the dimension personal distress, which is sometimes argued to measure self-management rather than empathy. Female indicates whether the participant is female (=1) or not (=0). Age gives the age of the participant. *, **, and *** document significance at the 5%, 1%, and 0.1% levels, respectively.
