# Supplementary material for: Empathy: A clue for prosocialty and driver of indirect reciprocity
Source: PLoS One. 2021 Aug 12;16(8):e0255071. doi: 10.1371/journal.pone.0255071 (PMC8360368; doi:10.1371/journal.pone.0255071)
Supplement: S2 Table — (PDF) [file pone.0255071.s002.pdf]

**S2 Table. Effect of another participant's empathy on expected prosocial behavior, type matching - Pooled OLS regression.**

| | Expected amount sent High empathy (1) | Expected amount sent Low empathy (2) | Expected amount sent Matched empathy (3) | Expected amount sent Non-matched empathy (4) |
|---|---|---|---|---|
| Empathy of other participant | 0.800*** | 0.768*** | 0.779*** | 0.785*** |
| | (0.078) | (0.075) | (0.074) | (0.065) |
| Same type of empathy (dictator & receiver) | 0.043 | 0.000 | | |
| | (0.172) | (0.102) | | |
| Own high empathy | | | -0.384 | -0.438 |
| | | | (0.359) | (0.349) |
| Constant | 0.204 | 0.749** | 0.729*** | 0.672* |
| | (0.182) | (0.284) | (0.240) | (0.354) |
| Observations | 230 | 315 | 281 | 264 |
| $R^2$ | 0.374 | 0.292 | 0.235 | 0.421 |

*Notes:* The table presents the results of a pooled OLS regression with robust standard errors clustered on the individual level in parentheses. The dependent variable is the expected amount sent measured as beliefs (stage 3). Empathy of other participant indicates every possible level of empathy the other participant can have. Same type of empathy indicates whether the dictator and recipient have the same type of empathy (=1) or not (=0) according to the following dummy categorization. High empathy is a dummy variable that categorizes the five levels of empathy into high empathy (=1) (very high and high empathy) and low empathy (=0) (very low, low, and medium empathy). Model 1 only considers participants with high empathy. Model 2 only considers participants with low empathy. Model 3 only considers observations for which the participant estimated the amount sent of another participant who had the same type of empathy. Model 4 only considers observations for which the participant estimated the amount sent of another participant who had the opposite type of empathy. *, **, and *** document significance at the 5%, 1%, and 0.1% levels, respectively.
