# Supplementary material for: Empathy: A clue for prosocialty and driver of indirect reciprocity
Source: PLoS One. 2021 Aug 12;16(8):e0255071. doi: 10.1371/journal.pone.0255071 (PMC8360368; doi:10.1371/journal.pone.0255071)
Supplement: S3 Table — (PDF) [file pone.0255071.s003.pdf]

S3 Table. Effect of the recipient's empathy on prosocial behavior, type matching - Pooled OLS regression.

	Amount sent High empathy (1)	Amount sent Low empathy (2)	Amount sent Matched empathy (3)	Amount sent Non-matched empathy (4)
Empathy of recipient	0.517***	0.397***	0.440***	0.457***
	(0.089)	(0.066)	(0.072)	(0.072)
Same type of empathy (dictator & receiver)	0.225	0.005		
	(0.172)	(0.092)		
Own high empathy			0.232	-0.030
			(0.399)	(0.411)
Constant	0.958***	1.381***	1.301***	1.108***
	(0.281)	(0.304)	(0.282)	(0.413)
Observations	230	315	281	264
R^2	0.163	0.072	0.111	0.107

Notes: The table presents the results of a pooled OLS regression with robust standard errors clustered on the individual level in parentheses. The dependent variable is the amount sent in the conditional dictator game (stage 4). Empathy of recipient indicates every possible level of empathy the recipient can have. Same type of empathy indicates whether the dictator and recipient have the same type of empathy (=1) or not (=0) according to the following dummy categorization. High empathy is a dummy variable that categorizes the five levels of empathy into high empathy (=1) (very high and high empathy) and low empathy (=0) (very low, low, and medium empathy). Model 1 only considers participants with high empathy. Model 2 only considers participants with low empathy. Model 3 only considers observations for which the participant sent money to another participant who had the same type of empathy. Model 4 only considers observations for which the participant sent money to another participant who had the opposite type of empathy. *, **, and *** document significance at the 5%, 1%, and 0.1% levels, respectively.
