# Supplementary material for: Empathy: A clue for prosocialty and driver of indirect reciprocity
Source: PLoS One. 2021 Aug 12;16(8):e0255071. doi: 10.1371/journal.pone.0255071 (PMC8360368; doi:10.1371/journal.pone.0255071)
Supplement: S4 Table — (PDF) [file pone.0255071.s004.pdf]

S4 Table. Effect of differences between the own amount sent and the expected amount sent of the counterpart on prosocial behavior.

	Amount sent (1)	Amount sent (2)
Difference own and expected amount sent	-0.006	
	(0.064)	
Absolute difference own and expected amount sent		-0.035
		(0.090)
Constant	2.586***	2.635***
	(0.176)	(0.232)
Observations	545	545
R^2	0.000	0.001

Notes: The table presents the results of a pooled OLS regression with robust standard errors clustered on the individual level in parentheses. The dependent variable is the amount sent in the conditional dictator game (stage 4). In Model 1 the independent variable is the difference between the own amount sent in the standard dictator game (stage 1) and the expected sending of the counterpart. In Model 2 the independet variable is the absolute difference between the own amount sent in the standard dictator game (stage 1) and the expected sending of the counterpart. *, **, and *** document significance at the 5%, 1%, and 0.1% levels, respectively.
