# Supplementary material for: Empathy: A clue for prosocialty and driver of indirect reciprocity
Source: PLoS One. 2021 Aug 12;16(8):e0255071. doi: 10.1371/journal.pone.0255071 (PMC8360368; doi:10.1371/journal.pone.0255071)
Supplement: S1 Appendix — (PDF) [file pone.0255071.s005.pdf]

S1 Appendix. Instructions

General instructions

General Instructions

Welcome to this study.

Please read the instructions carefully. All participants receive the same information:

- In this study, you will be paid in cash according to your decisions, and the decisions of the other participants.
- For arriving on time, you receive 4 CHF.
- All of the decisions, data and payments are anonymous, i.e. none of the participants learns how much money any other participant receives.
- The study consists of 4 parts and one questionnaire.
- With a probability of 50%, part 1 will be paid out and with a probability of 50%, part 4 will be paid out. Part 3 will be paid out for sure. There is no payment for part 2 and the questionnaire.
- You are not allowed to communicate with any of the other participants, to use a mobile phone, or to start other programs on the PC. If you break these rules, you will be excluded from the study and all payments.
- If you have any questions, please raise your hand. We will come to your place to answer your question.

Thank you very much for participating and enjoy!

Please read the instructions for part 1 on the next page.

Standard dictator game (stage 1)

Part 1

You will be assigned randomly to another participant. Neither you nor the other participant will ever learn about the identity of the other participant.

Your decision:

- You receive 10 CHF.
- The other participant does not receive any money.
- You decide if you want to transfer any of your 10 CHF, and if yes how much, to the other participant.
- You keep the money that you do not transfer to the other participant.
- The other participant receives the money that you transfer.

<u>Example 1:</u>

Suppose you make the following decision:

You can transfer up to 10 CHF to the **other participant**.
Please choose the amount you wish to transfer.

- ○ 0 CHF
- ○ 1 CHF
- ● 2 CHF
- ○ 3 CHF
- ○ 4 CHF
- ○ 5 CHF
- ○ 6 CHF
- ○ 7 CHF
- ○ 8 CHF
- ○ 9 CHF
- ○ 10 CHF

According to your decision, you transfer 2 CHF to the other participant and you keep 8 CHF for yourself.

Example 2:

Suppose you make the following decision:

You can transfer up to 10 CHF to the **other participant**.
Please choose the amount you wish to transfer.

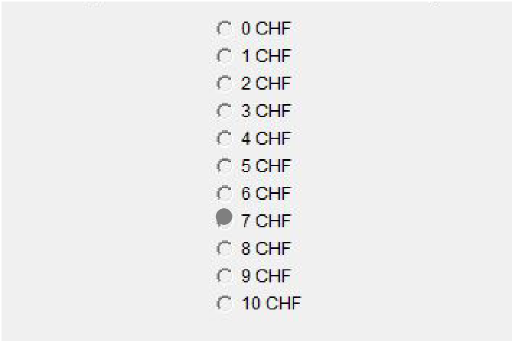

According to your decision, you transfer 7 CHF to the other participant and you keep 3 CHF for yourself.

Please answer two comprehension questions on your computer screen. Afterward, you can make your decisions.

If you read the instructions for part 1 and do not have any open questions, please click «Continue» on your computer screen.

Interpersonal Reactivity Index (IRI) - original scale (stage 2)

Extra sheet

		Never (1)	Seldom (2)	Sometimes (3)	Often (4)	Always (5)
1	I often have tender, concerned feelings for people less fortunate than me.	Never (1)	Seldom (2)	Sometimes (3)	Often (4)	Always (5)
2	I really get involved with the feelings of the characters in a novel.	Never (1)	Seldom (2)	Sometimes (3)	Often (4)	Always (5)
3	In emergency situations, I feel apprehensive and ill-at-ease.	Never (1)	Seldom (2)	Sometimes (3)	Often (4)	Always (5)
4	I try to look at everybody's side of a disagreement before I make a decision.	Never (1)	Seldom (2)	Sometimes (3)	Often (4)	Always (5)
5	When I see someone being taken advantage of, I feel kind of protective towards them.	Never (1)	Seldom (2)	Sometimes (3)	Often (4)	Always (5)
6	I sometimes feel helpless when I am in the middle of a very emotional situation.	Never (1)	Seldom (2)	Sometimes (3)	Often (4)	Always (5)
7	After seeing a play or movie, I have felt as though I were one of the characters.	Never (1)	Seldom (2)	Sometimes (3)	Often (4)	Always (5)
8	Being in a tense emotional situation scares me.	Never (1)	Seldom (2)	Sometimes (3)	Often (4)	Always (5)
9	I am often quite touched by things that I see happen.	Never (1)	Seldom (2)	Sometimes (3)	Often (4)	Always (5)
10	I believe that there are two sides to every question and try to look at them both.	Never (1)	Seldom (2)	Sometimes (3)	Often (4)	Always (5)
11	I would describe myself as a pretty soft-hearted person.	Never (1)	Seldom (2)	Sometimes (3)	Often (4)	Always (5)
12	When I watch a good movie, I can very easily put myself in the place of a leading character.	Never (1)	Seldom (2)	Sometimes (3)	Often (4)	Always (5)
13	I tend to lose control during emergencies.	Never (1)	Seldom (2)	Sometimes (3)	Often (4)	Always (5)
14	When I'm upset at someone, I usually try to "put myself in his shoes" for a while.	Never (1)	Seldom (2)	Sometimes (3)	Often (4)	Always (5)
15	When I am reading an interesting story or novel, I imagine how I would feel if the events in the story were happening to me.	Never (1)	Seldom (2)	Sometimes (3)	Often (4)	Always (5)
16	Before criticizing somebody, I try to imagine how I would feel if I were in their place.	Never (1)	Seldom (2)	Sometimes (3)	Often (4)	Always (5)

Average answers:	
Never:	1.00 – 1.49
Seldom:	1.50 – 2.49
Sometimes:	2.50 – 3.49
Often:	3.50 – 4.49
Always:	4.50 – 5.00

Interpersonal Reactivity Index (IRI) - reversed scale (stage 2)

Extra sheet

		Never (5)	Seldom (4)	Sometimes (3)	Often (2)	Always (1)
1	I often have tender, concerned feelings for people less fortunate than me.	Never (5)	Seldom (4)	Sometimes (3)	Often (2)	Always (1)
2	I really get involved with the feelings of the characters in a novel.	Never (5)	Seldom (4)	Sometimes (3)	Often (2)	Always (1)
3	In emergency situations, I feel apprehensive and ill-at-ease.	Never (5)	Seldom (4)	Sometimes (3)	Often (2)	Always (1)
4	I try to look at everybody's side of a disagreement before I make a decision.	Never (5)	Seldom (4)	Sometimes (3)	Often (2)	Always (1)
5	When I see someone being taken advantage of, I feel kind of protective towards them.	Never (5)	Seldom (4)	Sometimes (3)	Often (2)	Always (1)
6	I sometimes feel helpless when I am in the middle of a very emotional situation.	Never (5)	Seldom (4)	Sometimes (3)	Often (2)	Always (1)
7	After seeing a play or movie, I have felt as though I were one of the characters.	Never (5)	Seldom (4)	Sometimes (3)	Often (2)	Always (1)
8	Being in a tense emotional situation scares me.	Never (5)	Seldom (4)	Sometimes (3)	Often (2)	Always (1)
9	I am often quite touched by things that I see happen.	Never (5)	Seldom (4)	Sometimes (3)	Often (2)	Always (1)
10	I believe that there are two sides to every question and try to look at them both.	Never (5)	Seldom (4)	Sometimes (3)	Often (2)	Always (1)
11	I would describe myself as a pretty soft-hearted person.	Never (5)	Seldom (4)	Sometimes (3)	Often (2)	Always (1)
12	When I watch a good movie, I can very easily put myself in the place of a leading character.	Never (5)	Seldom (4)	Sometimes (3)	Often (2)	Always (1)
13	I tend to lose control during emergencies.	Never (5)	Seldom (4)	Sometimes (3)	Often (2)	Always (1)
14	When I'm upset at someone, I usually try to "put myself in his shoes" for a while.	Never (5)	Seldom (4)	Sometimes (3)	Often (2)	Always (1)
15	When I am reading an interesting story or novel, I imagine how I would feel if the events in the story were happening to me.	Never (5)	Seldom (4)	Sometimes (3)	Often (2)	Always (1)
16	Before criticizing somebody, I try to imagine how I would feel if I were in their place.	Never (5)	Seldom (4)	Sometimes (3)	Often (2)	Always (1)

Average answers:	
Never:	5.00 – 4.50
Seldom:	4.49 – 3.50
Sometimes:	3.49 – 2.50
Often:	2.49 – 1.50
Always:	1.49 – 1.00

Belief elicitation (stage 3)

Part 3

In part 3, you can earn money again. Your task is to guess how another participant decided in part 1 (see instructions for part 1). You will not learn the identity of the other participant and the other participant will not learn your identity. The other participant is a different person than the one you played with in part 1.

Reminder:

Decision in part 1:

How much of your 10 CHF do you want to transfer to the other participant?

Your payment:

- You receive **10 CHF** if you **correctly guess** the amount of money the other participant transferred in part 1.
- You receive **5 CHF** if your guess **differs by only 1 CHF** from the amount of money the other participant transferred in part 1.
- You receive **0 CHF** if your guess **differs by more than 1 CHF** from the amount of money the other participant transferred in part 1.

Link to the survey in part 2:

- Please take a closer look at the survey that all participants answered in part 2 of the study (see extra sheet). In the survey, you were asked how strongly you agree with 16 statements on a scale from 1 (Never) to 5 (Always).
- This scale allows us to calculate the average answer of every participant.

Example 1:

Assume a participant answered 8 times with 1 (Never) and 8 times with 2 (Seldom). The average answer is:

$\frac{8*1+8*2}{16}$ **= 1.5 → Never**

Example 2:

Assume a participant answered 6 times with 3 (Sometimes) and 10 times with 5 (Always). The average answer is:

$\frac{6*3+10*5}{16}$ **= 4.25 → Often**

- To guess how much money the other participant transferred in part 1, you can consider her average answer from the survey in part 2.
- However, at the time of your decision, you do not know the average answer of the other participant, because the random assignment to another participant takes only place at the end of the study.
- Thus, we ask you to guess how much money the other participant transferred in part 1 for every possible average answer in the survey.

Payment procedure:

- You will be assigned randomly to another participant.
- We will calculate the other participant's average answer in the survey of part 2.
- Using this average answer, we will compare your guess with the actual behavior of the other participant.

Example:

The following five screenshots show the input mask for part 3. For each of the 5 possible average answers of another participant in the survey, you guess how much money the other participant transferred in part 1.

1. Suppose the **average answer** of the other participant from the survey of part 2 is **Never (1.00-1.49).**
What do you think how much money she **transferred** in part 1?

○ 0 CHF
○ 1 CHF
○ 2 CHF
○ 3 CHF
● 4 CHF
○ 5 CHF
○ 6 CHF
○ 7 CHF
○ 8 CHF
○ 9 CHF
○ 10 CHF

2. Suppose the **average answer** of the other participant from the survey of part 2 is **Seldom (1.50-2.49).**
What do you think how much money she **transferred** in part 1?

○ 0 CHF
○ 1 CHF
○ 2 CHF
○ 3 CHF
● 4 CHF
○ 5 CHF
○ 6 CHF
○ 7 CHF
○ 8 CHF
○ 9 CHF
○ 10 CHF

3. Suppose the **average answer** of the other participant from the survey of part 2 is **Sometimes (2.50-3.49).**
What do you think how much money she **transferred** in part 1?

○ 0 CHF
○ 1 CHF
○ 2 CHF
○ 3 CHF
○ 4 CHF
● 5 CHF
○ 6 CHF
○ 7 CHF
○ 8 CHF
○ 9 CHF
○ 10 CHF

4. Suppose the **average answer** of the other participant from the survey of part 2 is **Often (3.50-4.49).**
What do you think how much money she **transferred** in part 1?

- 0 CHF
- 1 CHF
- 2 CHF
- 3 CHF
- 4 CHF
- 5 CHF
- 6 CHF
- 7 CHF
- 8 CHF
- 9 CHF
- 10 CHF

5. Suppose the **average answer** of the other participant from the survey of part 2 is **Always (4.50-5.00).**
What do you think how much money she **transferred** in part 1?

- 0 CHF
- 1 CHF
- 2 CHF
- 3 CHF
- 4 CHF
- 5 CHF
- 6 CHF
- 7 CHF
- 8 CHF
- 9 CHF
- 10 CHF

The following examples refer to the exemplary decisions on pages 3 and 4.

Example 1:
Suppose the actual average answer of the other participant in the survey of part 2 is **Seldom (1.50 – 2.49),** and in part 1 she transferred 4 CHF.
→ You receive **10 CHF**, because your guess is correct (see answer 2, p. 3).

Example 2:
Suppose the actual average answer of the other participant is **Often (3.50 - 4.49),** and in part 1 she transferred 6 CHF.
→ You **receive 5 CHF**, because your guess differs 1 CHF from the actual amount of money the other participant transferred (see answer 4, p. 4).

Example 3:
Suppose the actual average answer of the other participant is **Never (1.00 – 1.49)** and in part 1, she transferred 2 CHF.
→ You **receive no money**, because your guess differs more than 1 CHF from the amount of money the other participant transferred (see answer 1, p.3).

Please answer four comprehension questions on your computer screen. Afterward, you can make your guesses.

If you read the instructions for part 3 and do not have any open questions, please click «Continue» on your computer screen.

Conditional dictator game (stage 4)

Part 4

In part 4, we ask you to make a decision again. You will be assigned randomly to another participant in the study. You will not learn the identity of the other participant and the other participant will not learn your identity. The other participant is a different person than the one you played with in part 1 and part 3.

Your decision:

- The rules and decisions are the same as in part 1 of the study (see instructions for part 1).
- The only difference to part 1 is that you **can consider the average answer of the other participant** for your decision.

Execution of the payment:

- You are assigned randomly to another participant.
- We calculate the average answer of the other participant in the survey of part 2.
- Using this average answer, we implement your decision.
- At the time of your decision, you do not know the average answer of the other participant, because the random assignment takes only place at the end of the study.
- Thus, we ask you to decide how much money you want to transfer for every possible average answer of the other participant in the survey of part 2.

Please make your decisions on the computer screen.

If you read the instructions for part 4 and do not have any open questions, please click «Continue» on your computer screen.
